# COVID-19 vaccine hesitancy and social contact patterns in Pakistan: results from a national cross-sectional survey

**DOI:** 10.1186/s12879-023-08305-w

**Published:** 2023-05-11

**Authors:** Matthew Quaife, Sergio Torres-Rueda, Zlatina Dobreva, Kevin van Zandvoort, Christopher I. Jarvis, Amy Gimma, Wahaj Zulfiqar, Muhammad Khalid, Anna Vassall

**Affiliations:** 1 Faculty of Epidemiology and Population Health, London School of Hygiene and Tropical Medicine, London, UK; 2 Faculty of Public Health and Policy, London School of Hygiene and Tropical Medicine, London, UK; 3 Ministry of National Health Services Regulations and Coordination, Islamabad, Pakistan

**Keywords:** COVID-19, SARS-CoV2, Social contacts, Physical distancing, Vaccine perceptions

## Abstract

**Background:**

Vaccination is a key tool against COVID-19. However, in many settings it is not clear how acceptable COVID-19 vaccination is among the general population, or how hesitancy correlates with risk of disease acquisition. In this study we conducted a nationally representative survey in Pakistan to measure vaccination perceptions and social contacts in the context of COVID-19 control measures and vaccination programmes.

**Methods:**

We conducted a vaccine perception and social contact survey with 3,658 respondents across five provinces in Pakistan, between 31 May and 29 June 2021. Respondents were asked a series of vaccine perceptions questions, to report all direct physical and non-physical contacts made the previous day, and a number of other questions regarding the social and economic impact of COVID-19 and control measures. We examined variation in perceptions and contact patterns by geographic and demographic factors. We describe knowledge, experiences and perceived risks of COVID-19. We explored variation in contact patterns by individual characteristics and vaccine hesitancy, and compared to patterns from non-pandemic periods.

**Results:**

Self-reported adherence to self-isolation guidelines was poor, and 51% of respondents did not know where to access a COVID-19 test. Although 48.1% of participants agreed that they would get a vaccine if offered, vaccine hesitancy was higher than in previous surveys, and greatest in Sindh and Baluchistan provinces and among respondents of lower socioeconomic status. Participants reported a median of 5 contacts the previous day (IQR: 3–5, mean 14.0, 95%CI: 13.2, 14.9). There were no substantial differences in the number of contacts reported by individual characteristics, but contacts varied substantially among respondents reporting more or less vaccine hesitancy. Contacts were highly assortative, particularly outside the household where 97% of men's contacts were with other men. We estimate that social contacts were 9% lower than before the COVID-19 pandemic.

**Conclusions:**

Although the perceived risk of COVID-19 in Pakistan is low in the general population, around half of participants in this survey indicated they would get vaccinated if offered. Vaccine impact studies which do not account for correlation between social contacts and vaccine hesitancy may incorrectly estimate the impact of vaccines, for example, if unvaccinated people have more contacts.

**Supplementary Information:**

The online version contains supplementary material available at 10.1186/s12879-023-08305-w.

## Background

Over 750 million reported cases and 6.8 million deaths from COVID-19 have been recorded worldwide as of 2023 [1]. While most recorded cases and deaths initially occurred in high-income countries, a large proportion of the population of these countries is now vaccinated, and focus has increasingly turned to COVID-19 burden in low- and middle-income (LMIC) settings. Despite severe constraints in vaccine supply in LMIC settings, vaccination continues to play a key role in almost all countries’ COVID-19 response. For example, Pakistan – the focus of this study – has provided a range of COVID-19 vaccines developed by Pfizer, AstraZeneca, Sinovac, and Sinopharm, delivered through the public and private sectors [2]. As of 29 October 2021, a few months after the data collection forming the basis of this paper was completed, over 103 million vaccine doses had been administered to the country's 220 million population, and 40 million people were fully vaccinated (receiving one dose of one dose regimens, or two doses of two dose regimens) – a coverage of 18% [2]. Yet as supply has increased available vaccine stocks, substantial vaccine hesitancy has been observed worldwide, prompting a range of interventions to address hesitancy and encourage vaccination. In Pakistan, in July 2021 and in response to growing COVID-19 cases amid hesitancy, Sindh Province moved to block the SIM cards and social media accounts of unvaccinated residents.

Early in the pandemic, many countries introduced extreme physical distancing control measures to control SARS-CoV2 transmission [3]. Modelling studies suggested that without substantial mitigation measures, many LMIC settings, including South Asia, would experience a delayed, but severe epidemic [4, 5]. More recently, further modelling studies have been critical in informing vaccine introduction and rollout [6, 7]. Two pieces of data are critical for infectious disease models to give accurate projections of epidemic dynamics and vaccine impact: a) the number and nature of contacts which people have and which may lead to transmission, and b) the extent to which people will be hesitant in accessing vaccination. The association between social contacts and vaccine hesitancy is not well identified in the literature. This study aimed to collect and measure these data and explore their association in order to better inform policy responses, for example in recommending whether these factors should be accounted for in infectious disease modelling.

To accurately predict the likely impact of control measures, quantitative data on the number and type of contacts between people is required. To-date, few empirical studies have been published to assess the impact of COVID-19 control measures on contacts [8], and just one in South Asia [9, 10]. This lack of evidence means that SARS-CoV-2 transmission models for South Asia, including Pakistan, rely on synthetic contact matrices, which use demographic, household composition, classroom size and other data to adjust social contact data from primarily high-income settings [11, 12]. Vaccine hesitancy, defined by the SAGE working group as “delay in acceptance or refusal of vaccination despite availability of vaccination services” [13], has previously been shown to be higher in Pakistan than other settings [14, 15]. The working group’s Vaccine Hesitancy Determinants Matrix highlights “contextual, individual and group and vaccine/vaccination-specific influences”, which may vary at local levels (for example, the regions that feature in this analysis).

It is hypothesised that vaccine hesitancy and responsivity to other infectious disease control measures may be correlated. For example, the SAGE Working Group’s matrix identifies vaccine complacency as a driver of low vaccine uptake [13], potentially reflecting a perception of low risk from COVID-19 and a parallel insensitivity of behaviour to non-pharmaceutical control measures aiming to limit social contacts. To-date, no study has explored the relationship between social contacts and vaccine hesitancy. This is important as COVID-19 models which assume homogeneity in vaccine uptake and contact patterns may dramatically under- or over-estimate the impact and cost-effectiveness of social distancing and vaccine interventions, leading to incorrect policy decisions.

**Fig. 1:**
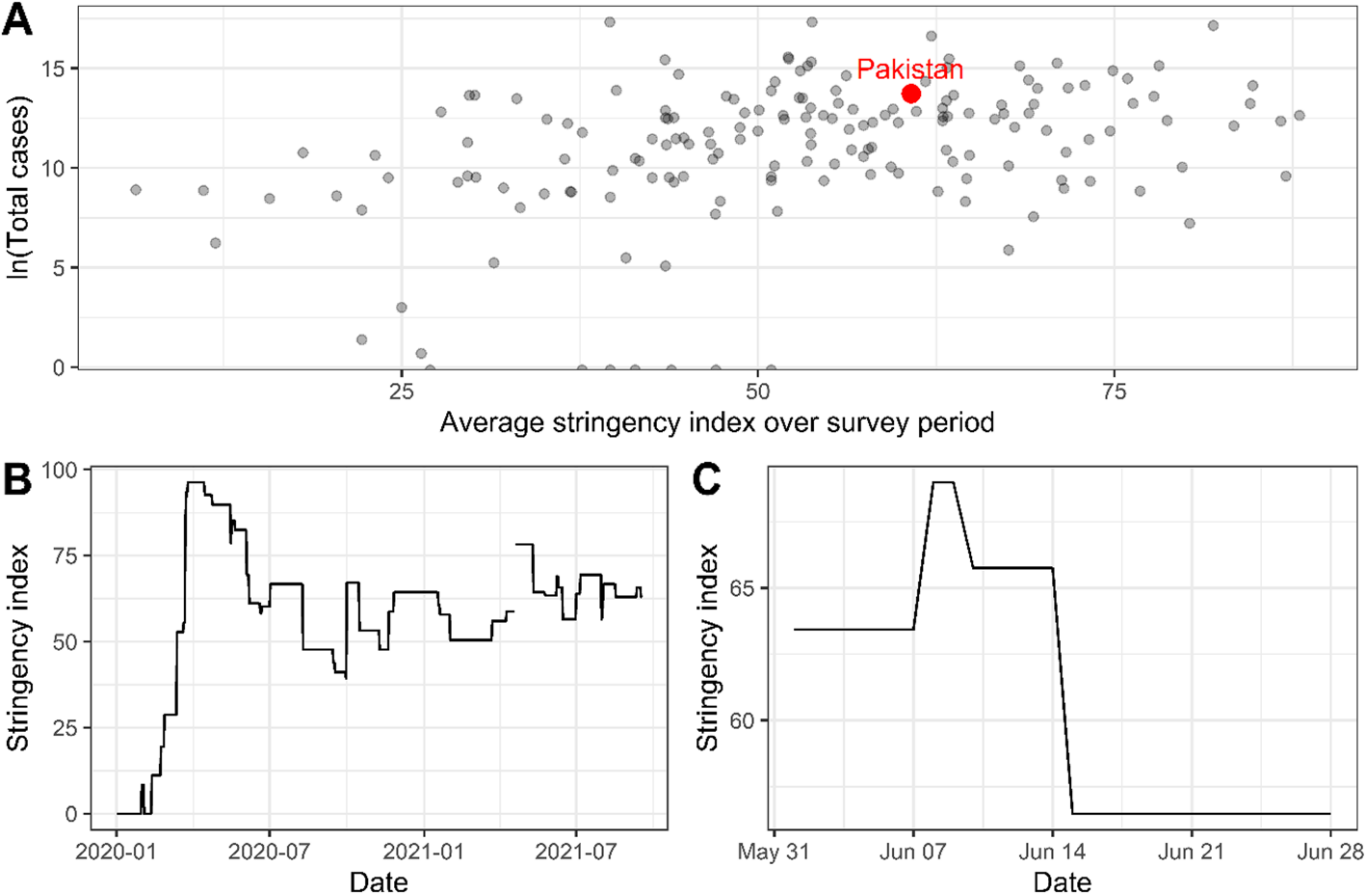
Stringency of COVID-19 control measures in Pakistan during COVID-19 pandemic. **A** shows ln(Total SARS-CoV-2 cases) on the first day of the survey, 29 May 2021, plotted against the average stringency index over the survey period. **B** plots Pakistan's stringency index over time during the COVID-19 pandemic. **C** plots the stringency index during the survey period, note that the scale of this panel differs from (**B**)

To date, the numbers of recorded cases and deaths in Pakistan are much lower than initial predictions, despite four clear epidemic waves of COVID-19 in the country and evidence elsewhere in the region of uncontrolled transmission requiring strong non-pharmaceutical intervention [1]. Pakistan's response was similar to other settings, implementing a range of restrictions including on gatherings, closure of educational and municipal buildings, and suspension of international flights and cross-border travel. As shown by the University of Oxford COVID-19 Government Response Tracker data in Fig. 1, these restrictions increased sharply in March 2020, decreased in intensity over subsequent months, and settled at a medium–high intensity in the period since. Panel A demonstrates that Pakistan's average stringency index [3] was in the top third of countries worldwide during the survey period, whilst panel C shows that there were few changes to the stringency of control measures during the survey period.

In a decentralised policy environment such as Pakistan, it is critical to understand how vaccination perceptions and hesitancy, and patterns of social contacts, vary across provinces to inform whether and how to implement different control measures alongside how to maximise vaccination coverage. In this study, we describe a nationally representative, province-stratified cross-sectional survey of vaccination perceptions and contact patterns among adults in Pakistan. We first describe the health and socioeconomic impacts of COVID-19 and control measures among the sample. We then summarise COVID-19 vaccine perceptions and hesitancy and variations across provinces and respondent characteristics. We also summarise how the quantity and type of social contacts vary across respondent characteristics.

## Methods

### Survey methodology

A geographically-stratified survey frame was devised for a face-to-face survey across four provinces – Baluchistan, Khyber Pakhtunkhwa, Punjab and Sindh – and Islamabad Capital Territory, where within-province recruitment was stratified across urban and rural areas based on relative population sizes across these areas within provinces. Data were collected between 31 May and 29 June 2021 by research assistants of IPSOS Mori, a market research consultancy.

A target sample size of 3,600 was devised based on the smallest within-province urban/rural area needed to detect a mean difference of 5 contacts between urban and rural areas at a confidence level of 95%, assuming 80% power based on the population variance of contacts across a recent LMIC social contact dataset. Primary sampling units were randomly selected proportionate to size, for example, cities in urban areas and villages in rural areas. In rural areas, secondary sampling units were randomly chosen at the village level and a well-known central landmark (e.g., mosque, shop, electricity transformer) was chosen in each, and four equally-sized quadrants were drawn in the area around this. Interviewers conducted five interviews in two alternate quadrants (e.g., 1 and 3). In urban areas, a similar important landmark was chosen in areas divided based on census data of around 250–270 households, and interviews recruited households using a random ballot of five households nearby and a three-household skip interval. One respondent was recruited per sampled household randomly chosen using a KISH grid, with interviews calling back up to three times if respondents were unavailable.

All face-to-face interviews were conducted in strict compliance with Pakistan government COVID-19 regulations. Participants were asked to choose a quiet, well-ventilated area for the interview to take place, and fieldworkers sanitised hands regularly, always maintained physical distance from participants, and did not shake hands. Respondents were asked a range of questions on COVID-19 including knowledge and experience of testing, isolation requirements and symptoms, alongside impacts on the household such as income or expenditure changes. Then, a short, validated vaccine hesitancy tool was shown to participants [15, 16], assessing respondent intentions to obtain a vaccine if one was offered alongside reasons for potential hesitancy, and a range of attitudinal questions asking respondents to designate their agreement with statements around safety, efficacy and importance of vaccination, for example, "vaccines are effective".

Respondents were then asked to report all direct physical and non-physical contacts made between 5am the day before and 5am the day of the survey (i.e., one 24 h period). A direct contact was defined as someone respondents met in person and with whom they had either i) "*Physical contact (any sort of skin-to-skin contact e.g. a handshake, embracing, kissing, sleeping on the same bed/mat/blanket, sharing a meal together out of the same bowl, playing football or other contact sports, sitting next to someone while touching shoulder to shoulder, *etc.*)*", or ii) "*Non-physical contact (you did not touch the person, but exchanged at least a few words, face-to-face within 2 m – for example, someone you bought something from in the market, or rode with on a public/private transportation vehicle, or worked with in the same area)"*. All respondents were over the age of 18 so no contact data were collected from children, however respondents were able to list contacts under the age of 18.

We made pragmatic adaptations to existing contact measurement tools to allow them to be conducted quickly face-to-face, to reduce respondent burden, and to ensure that aggregate contact data were not biased downwards by respondent fatigue. Respondents were first asked about contacts with members of their household the previous day, recording the contact age, gender, and whether contacts were physical or non-physical. Then respondents were asked how many non-household contacts they had had in the same timeframe. Those who reported nine or fewer outside household contacts were asked to describe each contact's age, gender, whether the contact was physical or non-physical, the duration of the contact, and whether a mask was worn by the respondent or contact. Those who reported ten or more outside-household contacts were asked how many of these contacts were physical/non-physical, whether they took place in school/work or elsewhere, and whether contacts were in the age ranges under 18, 18–59, or over 60. The complete survey tool, including these social contact questions, is shown in Additional file 1.

### Statistical analysis

R version 4.0.0 and Stata 15 were used for analyses; the code and data are publicly available. We use tables and descriptive plots to summarise participant characteristics, factors relating to the impact of COVID-19 and control measures on the household, and vaccine attitudes and hesitancy. We present exploratory disaggregations of these variables to consider variation by geographical and individual variables. We determine if there are important differences in mean contacts between groups by comparing mean contacts and use t-tests to determine the strength of differences.

For the social contacts analysis, we calculated the average number of social contacts per person per day, stratified by respondent age, sex, household size, socioeconomic status, province, and education level. We then calculated social contact matrices for the age-specific frequency of daily contacts, adjusting for contact reciprocity and the age distribution using national data [17]. We compared contact data with those of Pakistan in the synthetic matrices of Prem et al. [12]. As respondents under the age of 18 were not included as survey respondents, we imputed the average number of contacts of children with adults using the estimated total number of contacts of adults with children, adjusting for the national population demographics and assuming reciprocity on the total number of contacts. Contacts made between children were imputed using methods developed by Klepac et al. [18], and implemented in UK [19] and Kenyan studies [20]. This involved taking the ratio of the dominant eigenvalues between our matrices and comparable setting-adjusted matrices to scale the missing matrix elements for respondents under the age of 18.

### Ethics

Participation in the study was voluntary and analyses were conducted on anonymised data. The study was approved by the Observational/Interventions Research Ethics Committee of the London School of Hygiene and Tropical Medicine (Reference: 25,453).

## Results

### Respondent characteristics

In total, 3,658 people completed the survey with the intended 50/50 sample-level stratification across urban and rural areas and male and female respondents. In total, 8,211 interviews were attempted, 2,192 (27%) had no answer or no response, and there were 1,360 refusals to participate (17%). 56 interviews were interrupted (1%), 917 (12%) of respondents were not eligible for interview as stratification targets had already been met in that area, and 27 (0.3%) interviews removed due to quality control issues.

Table 1 shows that the age, gender and geographical distributions of respondents broadly reflect those of Pakistan, though the proportion of respondents aged over 55 years and 18–35 years were under-represented in our sample.

**Table 1:** Respondent characteristics in this study

	**Respondents in this survey (*n* = 3,658)**		**Pakistan national population projections**^a^
**Age group**			
*0–17*	0	-	
*18–25*	1,062	29%	19%^b^ +
*26–34*	1,092	30%	29% +
*35–44*	21	25%	20% +
*45–54*	423	11%	15% +
*55* +	160	4%	17% +
**Gender**			
*Male*	1,830	50%	51%
*Female*	1,828	50%	49%
**Province**			
*Baluchistan*	334	9%	5%
*Islamabad Capital Territory*	40	1%	1%
*Khyber Pakhtunkhwa*	624	17%	14%
*Punjab*	1,740	48%	57%
*Sindh*	920	25%	24%
**Urban/Rural**			
*Urban*	1,809	49%	52%
*Rural*	1,849	51%	48%

^a^National population projections obtained from Pakistan Bureau of Statistics and World Bank [17, 21]

^b^Represents population 20–25, as data unavailable for ages 18 and 19 years. + Proportion of adult population; adult proportions are therefore comparable across columns

### Implications of COVID-19 and control measures

**Fig. 2:**
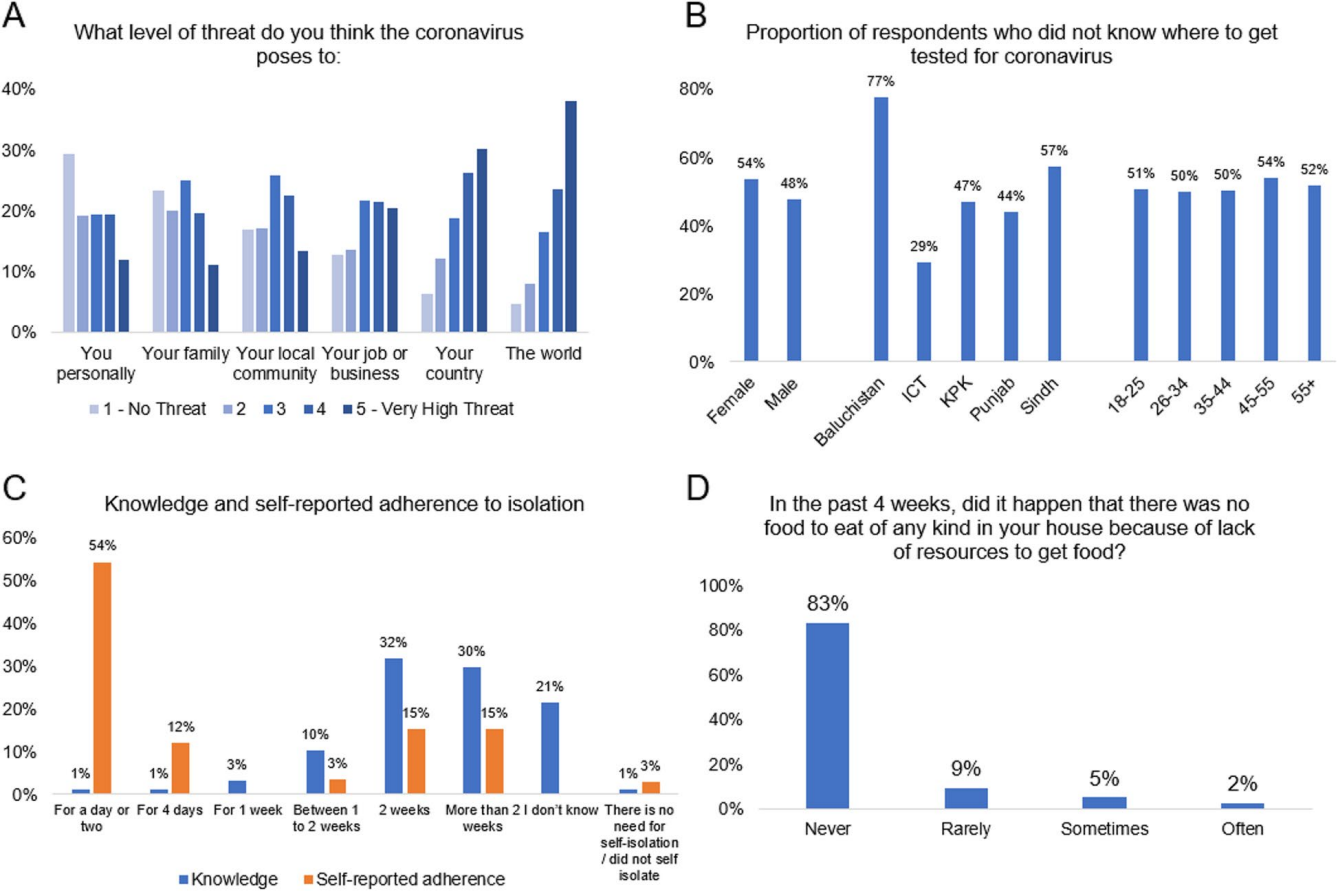
Description of (**A**) perceived threat from COVID-19 to respondents and groups to which they belong, **B** the proportion of respondents from different groups who reported not knowing where to obtain a COVID-19 test, **C** percentage of respondents reporting knowledge and self-reported adherence to self-isolation with COVID-19 symptoms, and (**D**) self-reported household hunger

Just 62 (1.6%) of participants reported ever obtaining a positive COVID-19 test. One-in-ten (365) reported perceiving a high risk of COVID-19 infection, while 45% perceived no or low risk that they would experience COVID-19. As in Fig. 2A, just 12% of respondents thought that COVID-19 posed a very high threat to them personally and 11% to their family; however, 30% thought COVID-19 posed a very high threat to the country and 38% to the world. Slightly more than half (51%) of respondents did not know where to obtain a COVID-19 test, with some variation by region and individual characteristics (Fig. 2B), and (308) 8% of respondents had ever taken a COVID-19 test. There was a clear gap between self-isolation knowledge and behaviours (Fig. 2C) – although 72% of respondents thought that after self-isolation was needed for longer than one week after coronavirus infection; 66% of the 62 respondents who had ever tested positive for COVID-19 reported self-isolating for four days or fewer.

Finally, 17% reported household hunger in the previous four weeks (Fig. 2D), with 60% of those experiencing hunger reported the primary reason was either household COVID-19 infection, COVID-19 control measures, or both. A substantial proportion of respondents were impacted in various ways: 25% of households experienced job losses, 30% of individuals spent working time home schooling or caring for other (Additional file 3, fig. 1A). Nearly one in five (19%) of respondents were able to work or study from home.

Respondents reported substantial economic insecurity due to COVID-19 and control measures. 77% reported a decrease in their income as a direct result of COVID-19 or control measures, of those 19% reported a decrease in income of more than half. Nearly all (94%) reported noticing an increase in prices in the past month, and 97% in the past year. To cope with income losses or increases in costs, 25% of respondents reported taking loans, 15% drawing from savings, 4% receiving gifts from family or friends, and 3% selling assets (Additional file 3, fig. 1B). On balance, the level of household spending across different items did not change substantially compared to before COVID-19 (Additional file 3, fig. 1C).

### Vaccine attitudes and hesitancy

Nearly half (48%) of participants agreed that they would get a vaccine if it were offered. Vaccine hesitancy was substantial and highest in Sindh and Baluchistan provinces where just 14% and 7% of respondents strongly agreed that they would get a vaccine if available (Additional file 3, fig. 2A). There was very strong evidence that hesitancy varied by socioeconomic status (Additional file 3, fig. 2B), for both strongly agreeing (non-parametric test for trend *p* < 0.001) and strongly disagreeing that they would get a vaccine (trend *p* < 0.001). There was weaker evidence of a trend by age groups (Additional file 3, fig. 2C) in strongly agreeing (trend *p* = 0.05) and strongly disagreeing that they would get a vaccine (trend *p* = 0.19), with older respondents substantially more likely to be willing to receive a vaccine. There were no substantial differences between genders.

Although 51% of all respondents thought vaccines were safe, 46% thought they were effective, and 47% thought they were important. These perceptions were much lower in Sindh and Baluchistan, the two provinces with lowest willingness to get a vaccine if available (Additional file 3, fig. 3). Among those reporting hesitancy in seeking vaccination, the primary reason was worry about side effects for 37% of respondents, whilst 16% thought vaccines were not effective, 15% thought that they were not at risk from COVID-19, and 13% were against vaccines in general. A further 10% cited time or cost, 4% lack of endorsement by religious leaders, and 3% fear of causing infertility.

### Contact patterns

**Fig. 3:**
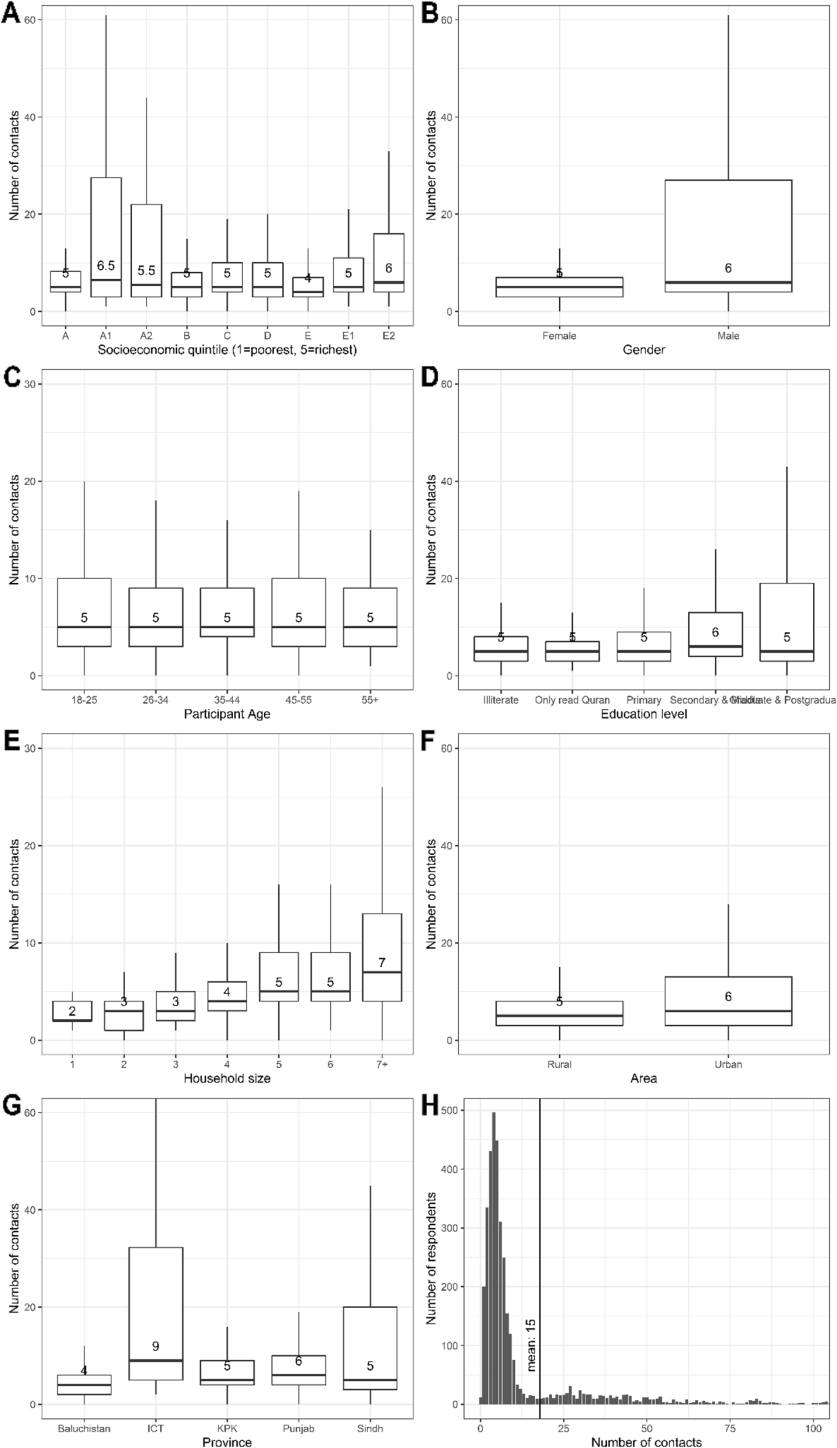
Median number of direct contacts (physical and non-physical) by (**A**) socioeconomic status, **B** gender, **C** respondent age, **D** education level, **E** household size, **F** living in an urban or rural area, and (**G**) province. Each panel shows the median, hinges (25th and 75th percentiles), and whiskers representing upper and lower adjacents. Outliers are not displayed in boxplots for scale, these are plotted in (**H**) showing the distribution of the number of direct contacts reported

In total, 56,455 contacts were reported, 14,786 (26%) of which were household contacts. One-in-three participants (29%) reported nine or fewer contacts, so we have full information on 16,357 contacts, 77% of which were household contacts. The mean number of contacts reported was 15 (median 5, IQR 3–9). Participants reported a mean of 4 household contacts (median 3, IQR 2–5) and 11 non-household contacts (median 2, IQR 0–4). As shown in Fig. 3, there was no substantial variation in the number of contacts by socioeconomic status, gender, participant age, education level, province, or whether the respondent lived in an urban or rural area. As expected, the number of contacts reported increased with household size as 35% (14,786/41,669) of contacts were reported within the household.

**Fig. 4:**
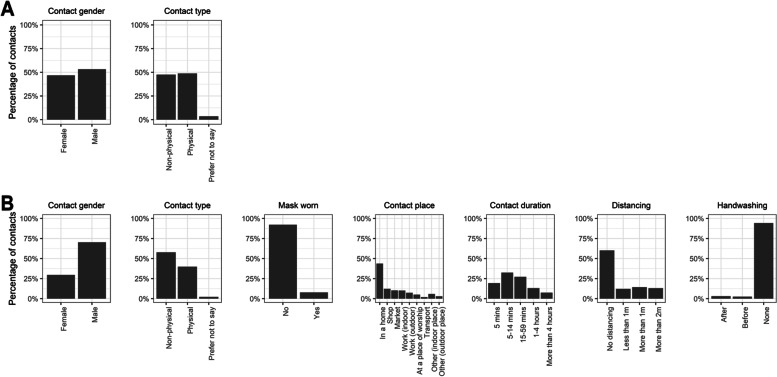
Characteristics of (**A**) household and (**B**) non-household contacts for which full information was gathered

Figure 4 summarises the characteristics of the 16,357 (28%) contacts for which we have detailed information as respondents reported fewer than ten contacts in each setting, comprising 12,540 (85%) of household contacts and 3,817 (9%) of non-household contacts. Within the household, roughly half of contacts were physical and equally split between genders. Outside of the household, 70% of all contactees were men. There was substantial assortativity by gender as men represented 97% of the outside household contacts of male respondents, compared to 29% of outside household contacts of female respondents. Assortativity by gender was lower for household contacts, where just 46% of male respondents' contacts were male and 59% of female respondents' contacts female (*p* < 0.001). Nearly half (49%) of male respondent's contacts were physical, compared to 44% of those of female respondents (*p* < 0.001); 92% of outside household contacts took place without masks being worn, and 43% took place in a residential property. Among those reporting detailed contacts, most (33%) contacts were between 5 and 14 min in length, with a further 27% between 15–59 min in length.

**Fig. 5:**
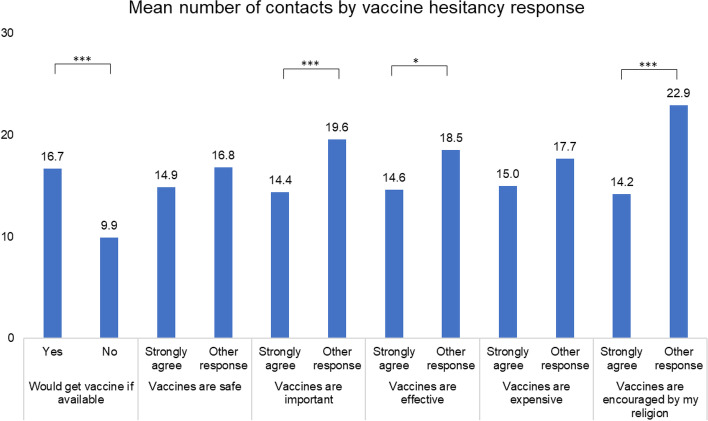
Mean number of contacts by those responding that they strongly agree with vaccine hesitancy questions. Asterisks represent difference in t-tests of mean contacts between groups: *** *p* < 0.01, ** *p* < 0.05, *p < 0.1

We explore how mean reported contacts vary across respondents reporting different extents of vaccine hesitancy in Fig. 5. First, we find that those strongly agreeing that they would get a vaccine report a greater number of contacts (16.7 compared to 9.9, t-test *p*-value < 0.01) which is beneficial to reducing cases through vaccination since reduction in disease risk from vaccination will counteract their having greater numbers of contacts. However, we find that people who strongly agree that vaccines have positive traits have significantly fewer contacts, specifically that vaccines are important (14.4 compared to 19.6, *p* = 0.01), effective (14.6 compared to 18.5, *p* = 0.07), or encouraged by their religion (14.2 compared to 22.9, *p* < 0.01). These sources of hesitancy may be important since lower vaccination uptake among those with more contacts will mean existing models will overestimate the impact of vaccination.

**Fig. 6:**
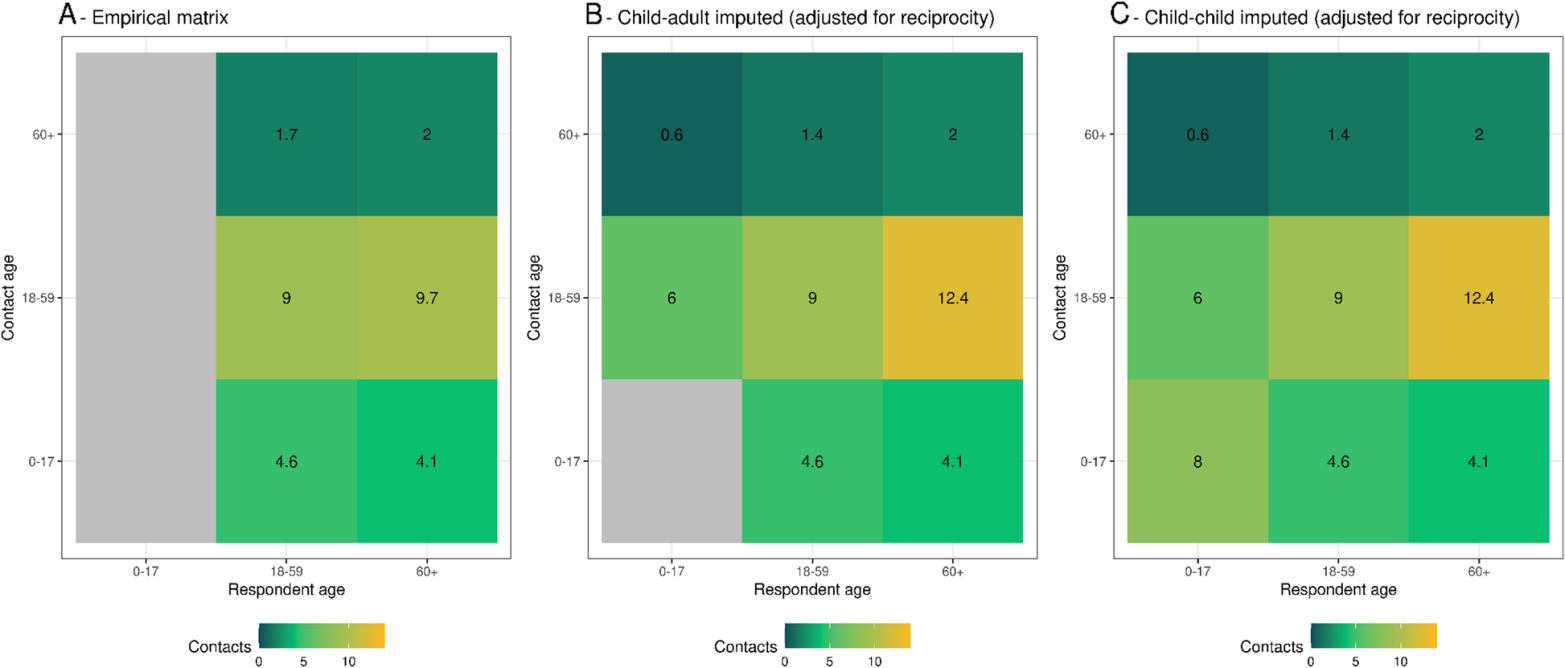
Age-stratified mean number of reported contacts from survey respondents, where (**A**) is the unadjusted contact matrix, **B** the mixing matrix produced when estimates from Prem et al. are used to impute contacts between children, and reported adult–child contacts are used to impute child–adult contacts**,** and (**C**) the mixing matrix produced when matrix (**B**) is adjusted for reciprocity using the age structure of Pakistan in 2020

Figure 6 shows age-specific contact matrices, where panel A shows an asymmetric matrix directly estimated from all contacts in this study without adjusting for demography. Unadjusted location-specific contact matrices for all contacts and detailed contacts are shown in Additional file 3, figs. 4 and s5 respectively. Figure 5B uses pre-COVID-19 synthetic contact matrices for Pakistan to impute contacts between children, and panel C adjusts this imputed matrix for age distribution and symmetry. Compared to the synthetic matrices of Prem et al. [12], we estimate a 9% reduction in contacts in this study. Among contacts for which we have detailed data, we observe some evidence of age-assortativity (Additional file 3, fig. 5C) in non-household and household contacts, and parent–child interactions in the household.

## Discussion

In a nationally representative survey stratified by age, gender, and urban/rural areas within provinces, we present evidence of the impact of COVID-19 on households and report considerable vaccine hesitancy in some provinces. Respondents perceived COVID-19 as a threat, but not to themselves – 45% of respondents perceived that they were at no or low risk from COVID-19. Furthermore, although 72% of respondents knew that self-isolation was required for longer than one week, 66% of those receiving positive COVID-19 tests self-isolated for four days or fewer. Just over half (51%) of respondents did not know where to obtain a COVID-19 test. The economic impact of COVID-19 and control measures appears to have been reasonably severe. Finally, we show that social contacts are highly assortative by gender and are estimate to have reduced in number since before the COVID-19 pandemic by 9%.

Although around half of respondents indicated that they would obtain a vaccine if available we saw considerable vaccine hesitancy, particularly in Baluchistan and Sindh provinces. Previous vaccine hesitancy surveys in Pakistan found that confidence in the importance, safety, and effectiveness of vaccines fell between 2015 and 2019 [15], with controversy around polio vaccination postulated as a potential reason [22]. This study's findings align with other vaccine hesitancy studies in Pakistan, though we find a slightly lower acceptability of vaccination compared to surveys of the general population [23] and health workers [14]. Compared to 2020 estimates using the same survey questions [15], we find respondents were much less likely to strongly agree that vaccines are effective, safe, and important – reductions of 73%, 54% and 70% respectively.

We find strong evidence that contacts are highly gender assortative, and 97% of male outside household contacts were with other men. We also see some evidence of age assortiveness outside household contacts, though we were only able to explore this in respondents who report fewer than ten outside household contacts. Like many other countries, Pakistan implemented a range of non-pharmaceutical interventions in 2020 and 2021 in response to increases in COVID-19 cases. When this survey was conducted in June 2021, many of these had been relaxed, however the average COVID-19 restriction stringency index in the survey period was 61 out of 100. We do not observe a reduction in contacts as seen in other settings – systematic review evidence indicates that contacts reduced by 62–83% under COVID-19 restrictions on average, though we note many of these were conducted earlier in the pandemic and under more intensive non-pharmaceutical interventions [8]. Without pre-COVID-19 empirical contact data from Pakistan, we had to compare our estimates to synthetic (modelled) contact estimates, which may be biased. Social contact studies such as this are critical to understand the sensitivity of contacts and behaviour to interventions as they continue to be implemented and relaxed in different ways. It may also be the case that COVID-19 restrictions may be less effective in the long term in reducing social contacts than elsewhere, perhaps due to the substantial economic and personal impact of restrictions found here.

Our findings that the mean number of contacts differ significantly by willingness to accept vaccination and perceptions of vaccines are important, as they indicate that existing COVID-19 impact models may be incorrect in assuming homogeneity in both vaccine uptake and contact patterns. Although such models continue to be a powerful tool in motivating for resources to vaccinate populations worldwide [6, 7], adapting such models to incorporate correlation between disease risk, as defined by number of contacts, and vaccination attitudes could show the importance of targeting high-contact vaccine hesitant groups, for example.

Considerable food and economic vulnerability was reported due to COVID-19 or control measures. Over 77% of respondents reported a partial or complete loss of income, with one in five of those reporting income reductions of more than half. Almost all (94%) respondents perceived price increases in the previous month. In order to cope with these pressures, 25% of respondents reported taking loans and 15% drew on savings. Although the prevalence of COVID-19 was low, and these factors can largely be attributed to control measures rather than illness from COVID-19 itself, it is important to recognise the counterfactual of no control measures is an unmitigated epidemic, and not an absence of these harms. Stringent control measures which cause economic and food insecurity are not likely to be sustainable in the long term if not accompanied by social protection mechanisms.

These data were collected during a period of decline of COVID-19 cases in Pakistan, on the first day of the survey the seven day average number of cases was 2,425, which reduced to 935 on the last day of the survey – this was one of the lowest case rates between the country's third and fourth waves of infection. Collecting contact data in a period of decline in case rates may have affected results, for example capturing increased contact rates as people perceive lower disease risk from daily activities. Conversely, case rates may have decreased due to more stringent control measures which acted by reducing contact opportunities and rates.

This study has a number of limitations. To make the contact survey feasible for data collection as part of a wider survey on COVID-19 impacts and vaccination perceptions, we simplified the contact tool for respondents who reported ten or more outside household contacts. We therefore have very limited information outside of the age and location category of these contacts, and contacts reported in this way were a substantial proportion (71%) of the total sample. We note that in a previous study using this method of collecting aggregate contact data in Kenya [20], 70% of contacts were reported through aggregate questions. The main risk of bias from this may stem from respondents rounding up or down to anchor numbers (e.g. units of five), though although a few respondents cluster around 25 contacts, we don't see much evidence of this in Fig. 1H. Overall, the loss of granularity was beneficial to reducing respondent burden, but reduced comparability to other studies. The standard contact measurement approach – where data collections guide respondents through their day and ask for all contacts, remains the gold-standard measurement tool, and this method also allows more granular contactee age data, which is important to inform models. In addition, in a culture where even slight physical contact such as a handshake can be taboo between men and women, we may be at risk of contact under-reporting to an unknown extent without conducting further validation work.

Although the vaccine hesitancy tool we use has been widely implemented in almost all countries worldwide, we cannot be sure how much an intention-behaviour gap exists with respect to accepting a vaccine if offered, nor the correlation between responses to different hesitancy questions and real-world uptake. Finally, evidence is now clear that SARS-COV-2 transmission is predominantly airborne [24], meaning that the number of contacts is likely less important to understanding COVID-19 dynamics than the location, ventilation, proximity and length of contacts.

## Conclusion

Like other settings, Pakistan continues to implement and relax control measures in response to the COVID-19 pandemic. In this representative national survey we find evidence of substantial vaccine hesitancy, alongside high knowledge but poor adherence to self-isolation after COVID-19 infection. We find that social contacts reduced by 9% compared to estimates from before COVID-19, that contacts are highly assortative by gender and, to a lesser extent, age, and that vaccination attitudes are associated with disease risk through different contact patterns. This is the first study to measure social contact patterns after COVID-19 control measures have been implemented in South Asia. Large negative impacts of COVID-19 and control measures on economic and food security suggest increased social protection may be needed.

## Supplementary Information

**Additional** **file** **1.** Mixing data collection tool.

**Additional file 2.** Measurement of socioeconomic status, and food and economic security.

**Additional file 3.** Supplementary figures and additional results.

## Data Availability

Anonymised datasets and code available from corresponding author upon reasonable request.

## Abbreviations

COVID-19: Coronavirus disease 2019
LMIC: Low and middle income countries
SARS-CoV-2: Severe acute respiratory syndrome coronavirus-2
SAGE: Scientific Advisory Group for Emergencies
