# Supplementary figures and images for: COVID-19 vaccine hesitancy and social contact patterns in Pakistan: results from a national cross-sectional survey
Source: BMC Infect Dis. 2023 May 11;23:321. doi: 10.1186/s12879-023-08305-w (PMC10174611; doi:10.1186/s12879-023-08305-w)

# Additional file 1. Mixing data collection tool


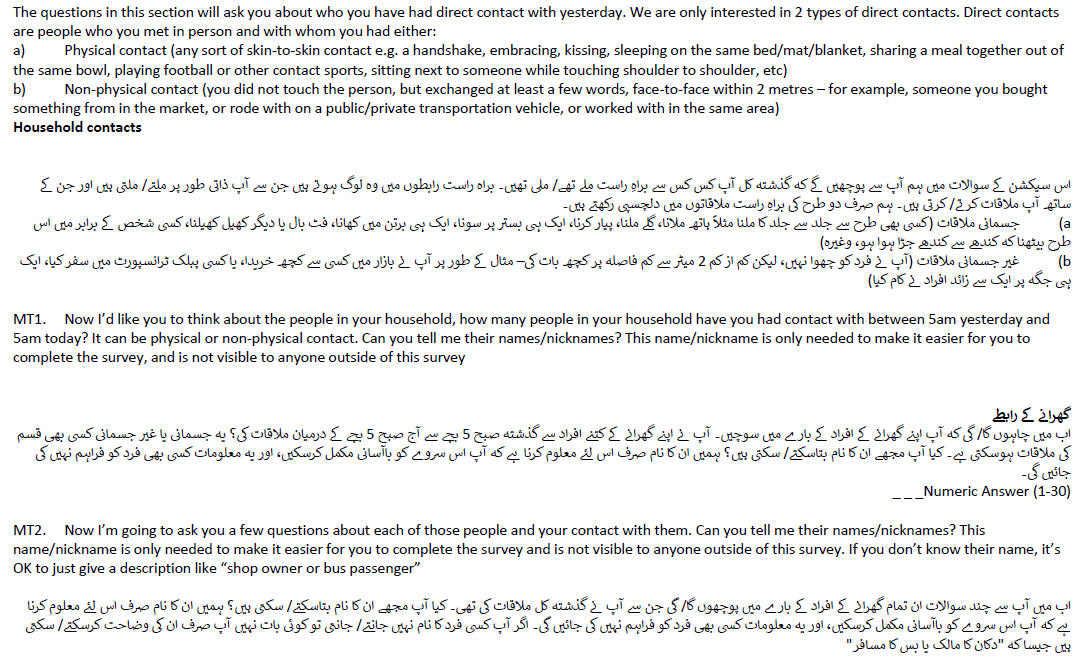


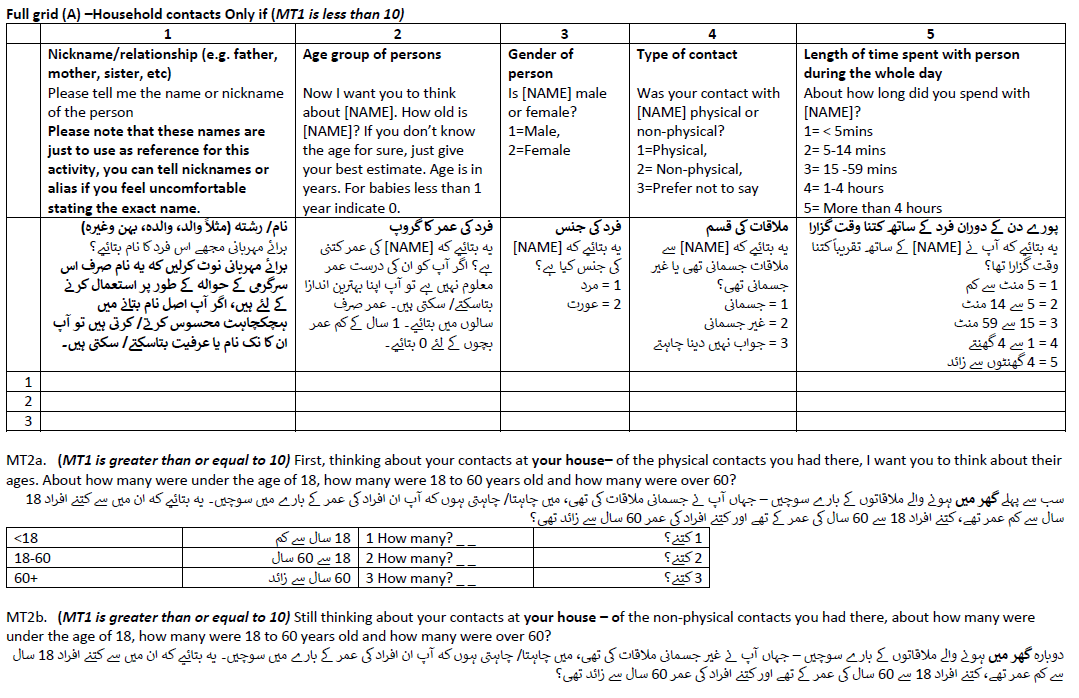


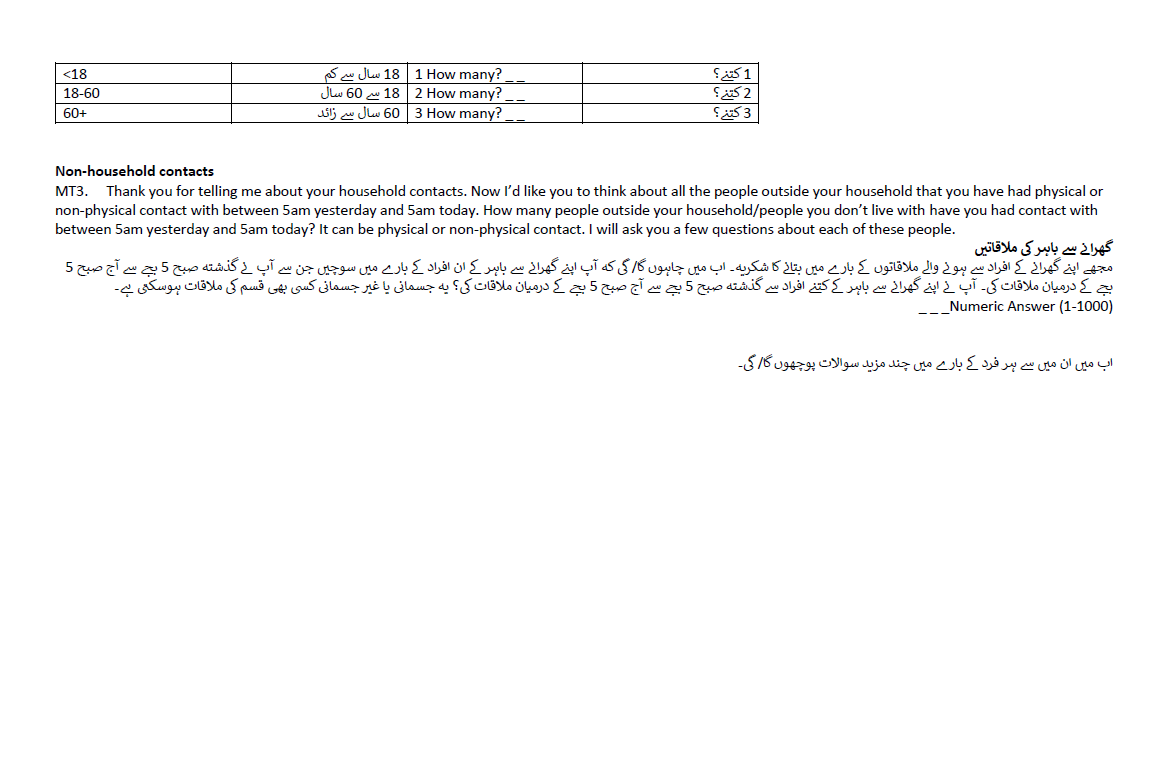


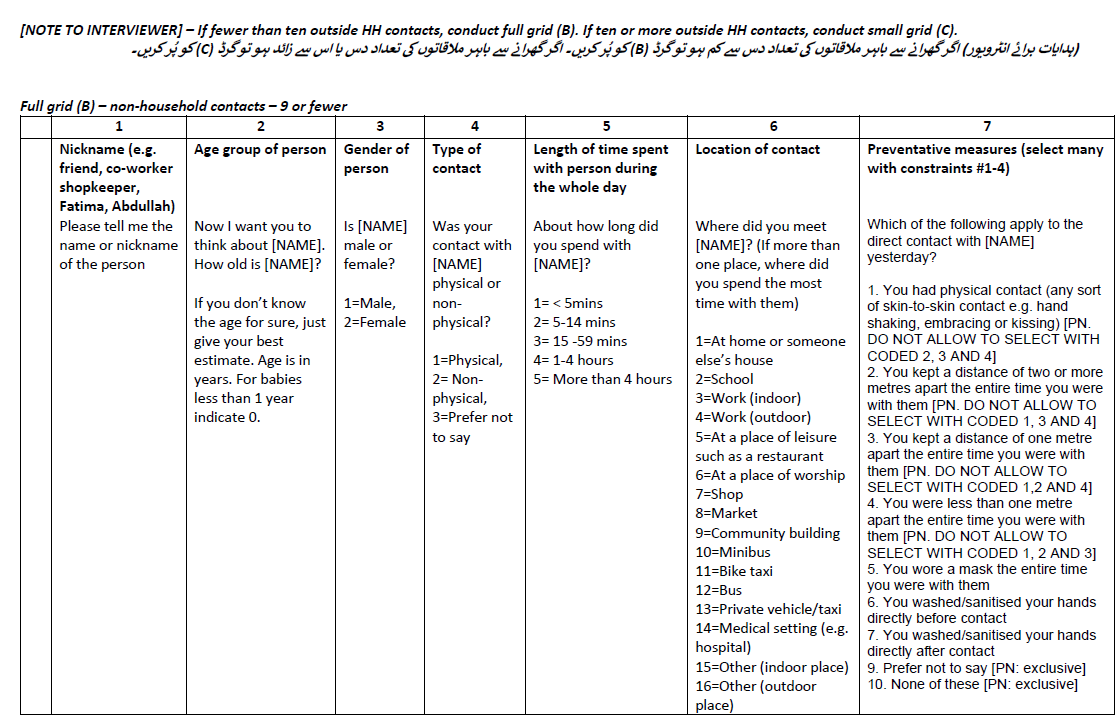


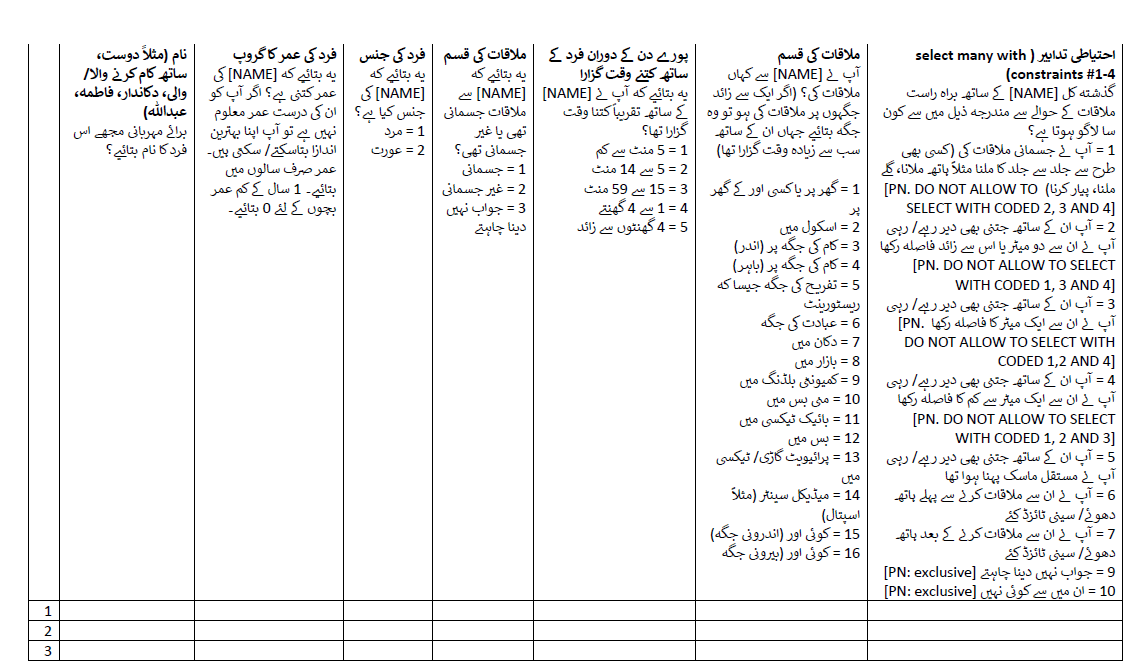


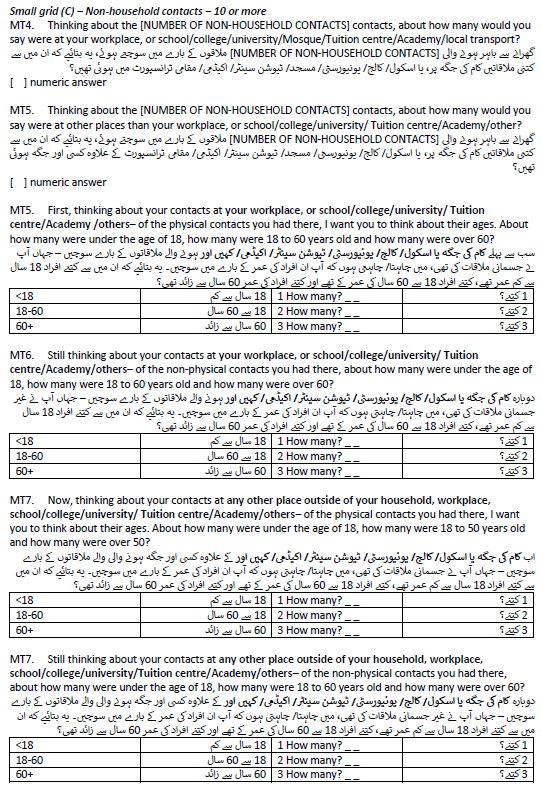

Supplement: Supplementary file 1 — Additional file 1. Mixing data collection tool. [file 12879_2023_8305_MOESM1_ESM.docx]
