# Supplementary material for: COVID-19 vaccine hesitancy and social contact patterns in Pakistan: results from a national cross-sectional survey
Source: BMC Infect Dis. 2023 May 11;23:321. doi: 10.1186/s12879-023-08305-w (PMC10174611; doi:10.1186/s12879-023-08305-w)
Supplement: Supplementary file 2 — Additional file 2. Measurement of socioeconomic status, and food and economic security. [file 12879_2023_8305_MOESM2_ESM.docx]

# Additional file 2. Measurement of socioeconomic status, and food and economic security


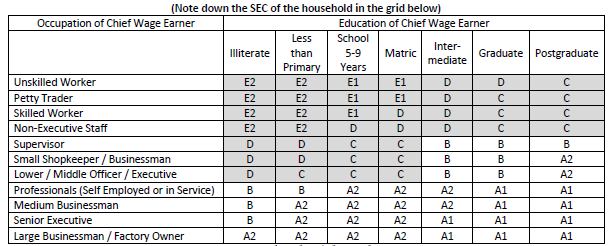


**Table 1: Definition of socioeconomic status**


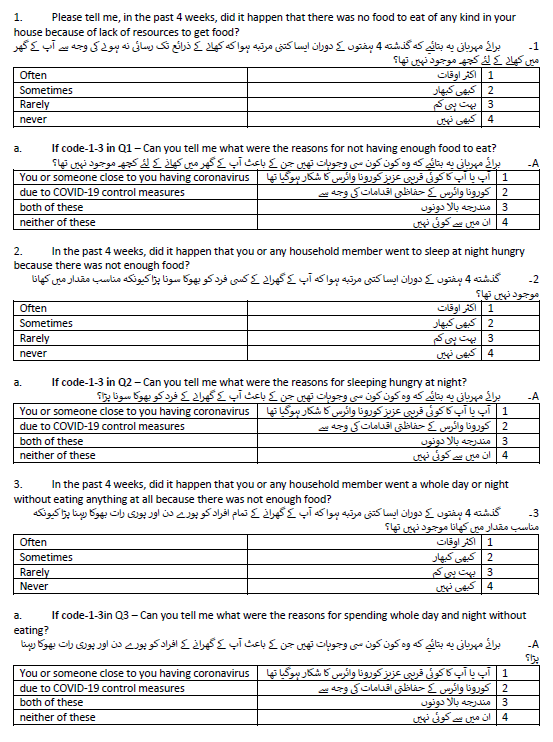


**Table 2: Household hunger scale**
