# Supplementary material for: COVID-19 vaccine hesitancy and social contact patterns in Pakistan: results from a national cross-sectional survey
Source: BMC Infect Dis. 2023 May 11;23:321. doi: 10.1186/s12879-023-08305-w (PMC10174611; doi:10.1186/s12879-023-08305-w)
Supplement: Supplementary file 3 — Additional file 3. Supplementary figures and additional results. [file 12879_2023_8305_MOESM3_ESM.docx]

# Additional file 3. Supplementary figures and additional results


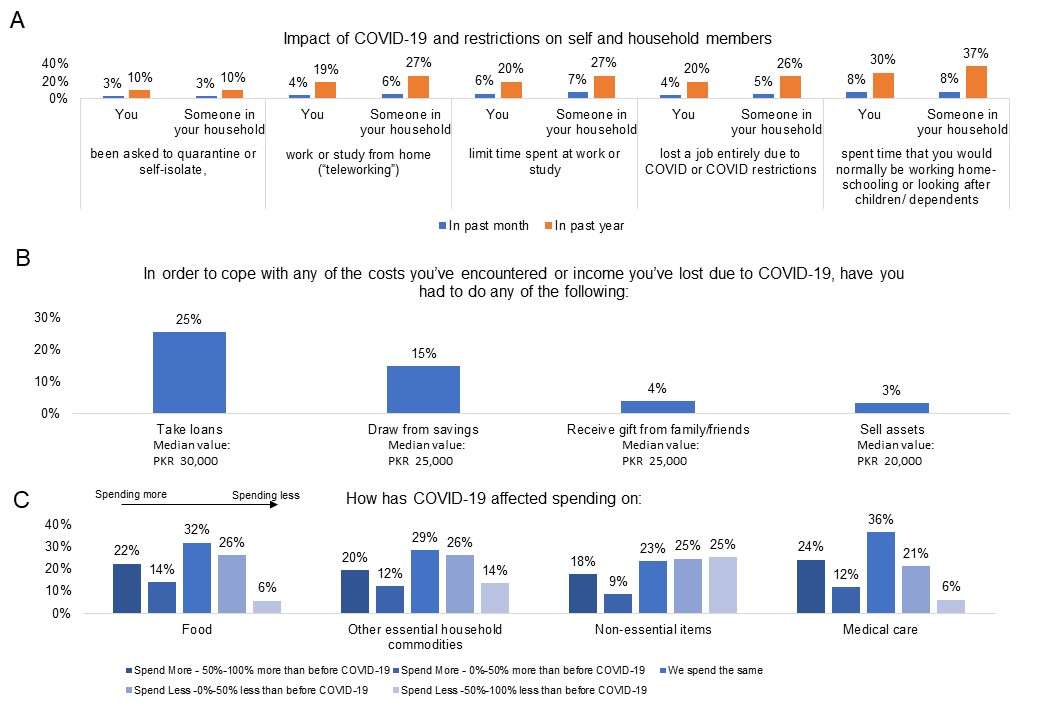


**Additional file 3. Figure 1**


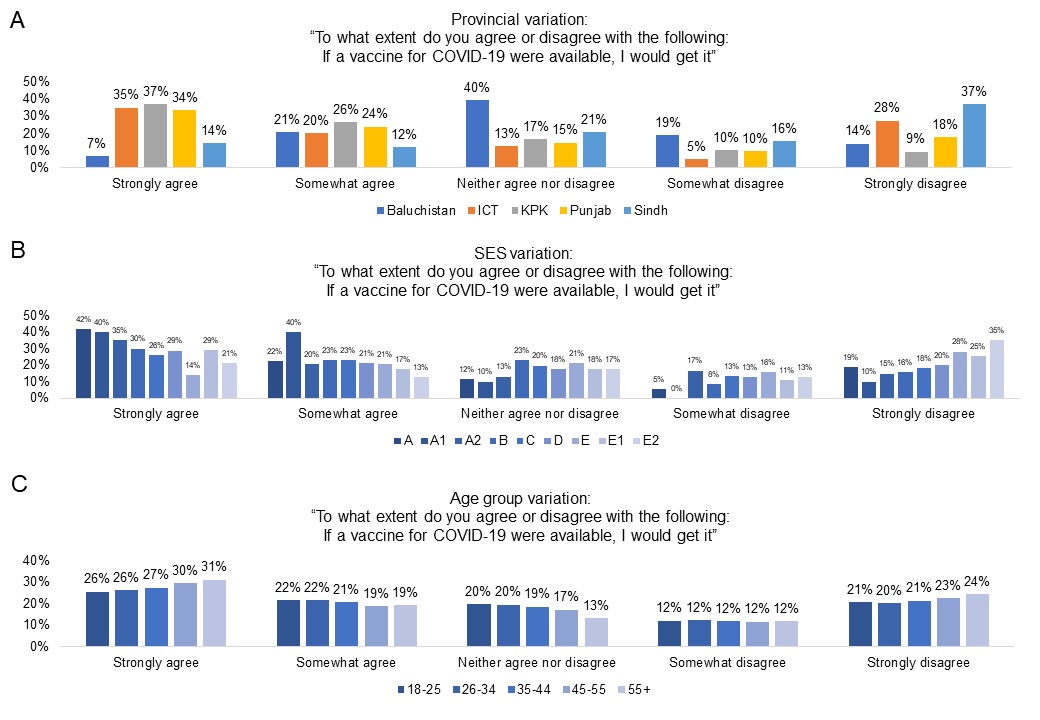


**Additional file 3. Figure 2**


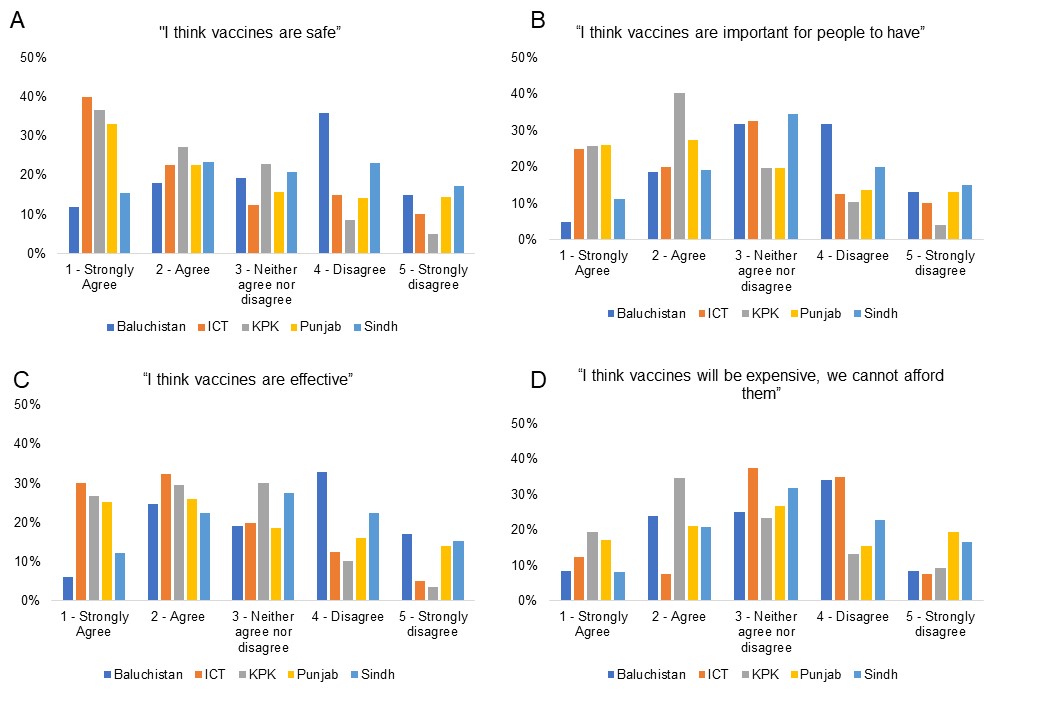


**Additional file 3. Figure 3**


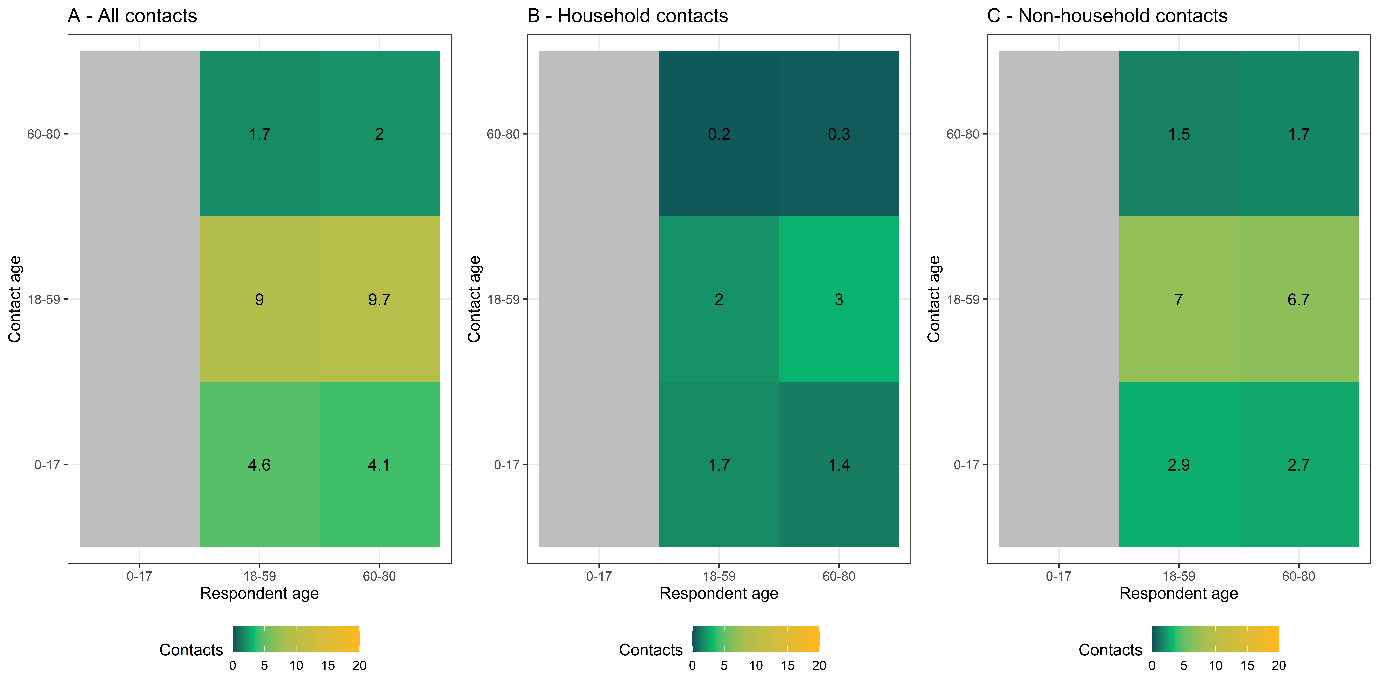


**Additional file 3. Figure 4**


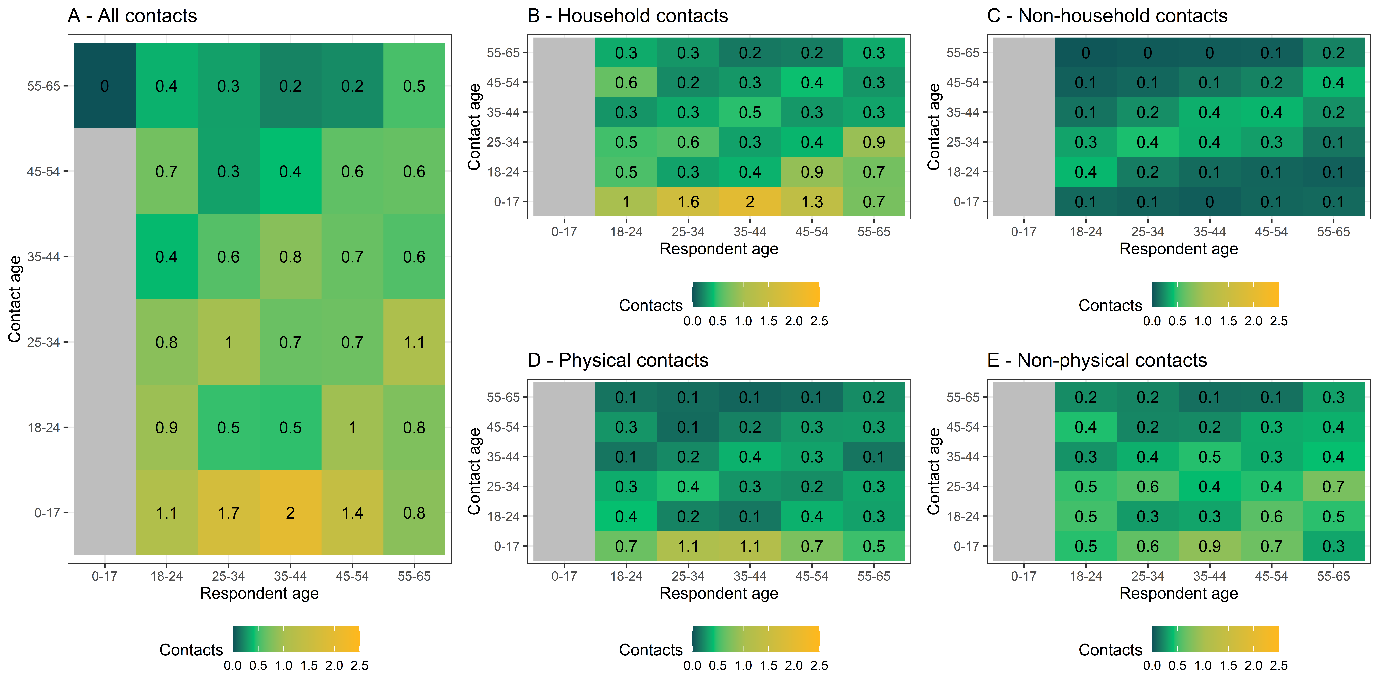


**Additional file 3. Figure 5**
